# NTRC Effects on Non-Photochemical Quenching Depends on PGR5

**DOI:** 10.3390/antiox10060900

**Published:** 2021-06-03

**Authors:** Belen Naranjo, Jan-Ferdinand Penzler, Thilo Rühle, Dario Leister

**Affiliations:** Plant Molecular Biology, Faculty of Biology, Ludwig-Maximilians-Universität München, D-82152 Planegg-Martinsried, Germany; JanFerdinand.Penzler@biologie.uni-muenchen.de (J.-F.P.); thilo.ruehle@biologie.uni-muenchen.de (T.R.)

**Keywords:** Arabidopsis, thioredoxins, NTRC, PGR5, redox regulation, cyclic electron flow, non-photochemical quenching, light stress, plant acclimation

## Abstract

Non-photochemical quenching (NPQ) protects plants from the detrimental effects of excess light. NPQ is rapidly induced by the trans-thylakoid proton gradient during photosynthesis, which in turn requires PGR5/PGRL1-dependent cyclic electron flow (CEF). Thus, *Arabidopsis thaliana* plants lacking either protein cannot induce transient NPQ and die under fluctuating light conditions. Conversely, the NADPH-dependent thioredoxin reductase C (NTRC) is required for efficient energy utilization and plant growth, and in its absence, transient and steady-state NPQ is drastically increased. How NTRC influences NPQ and functionally interacts with CEF is unclear. Therefore, we generated the *A. thaliana* line *pgr5 ntrc*, and found that the inactivation of PGR5 suppresses the high transient and steady-state NPQ and impaired growth phenotypes observed in the *ntrc* mutant under short-day conditions. This implies that NTRC negatively influences PGR5 activity and, accordingly, the lack of NTRC is associated with decreased levels of PGR5, possibly pointing to a mechanism to restrict upregulation of PGR5 activity in the absence of NTRC. When exposed to high light intensities, *pgr5 ntrc* plants display extremely impaired photosynthesis and growth, indicating additive effects of lack of both proteins. Taken together, these findings suggest that the interplay between NTRC and PGR5 is relevant for photoprotection and that NTRC might regulate PGR5 activity.

## 1. Introduction

Plants are sessile organisms and need to cope with continuous changes in light intensity by adjusting energy utilization to metabolic requirements and the amount of light available, which in turn necessitates the constant regulation of photosynthesis. During the photosynthetic process, light energy is utilized to extract electrons from water, which are transferred through the thylakoid membrane of the chloroplasts during linear electron flow (LEF) to ultimately reduce NADP^+^ to NADPH. LEF involves photosystem II (PSII), the cytochrome (cyt) *b_6_f* complex and photosystem I (PSI). During this process, a transmembrane proton gradient is generated, which is required for ATP synthesis, but also provides protection against damage caused by excess light. The resulting acidification of the thylakoid lumen downregulates the activity of the cyt *b_6_f* complex and triggers the thermal dissipation of the excess energy at PSII, a phenomenon known as non-photochemical quenching (NPQ) [1]. Both effects decrease levels of LEF, and consequently protect the two photosystems against overreduction and photodamage.

In addition to LEF, alternative thylakoid electron pathways exist, including cyclic electron flow (CEF) around PSI, which effectively returns electrons derived from PSI to the plastoquinone (PQ) pool and the cyt *b_6_f* complex. Like LEF, CEF contributes to the formation of the proton gradient across the thylakoid membrane, but without the net production of NADPH. Therefore, CEF allows the ATP/NADPH ratio to be adjusted and plays an important role in photoprotection [2,3,4]. Two different CEF pathways have been described: (i) the NADPH dehydrogenase-like (NDH) complex-dependent pathway [5,6,7,8] and (ii) the “antimycin A (AA)-sensitive pathway” which depends on the protein pair PGR5/PGRL1 [9,10,11,12]. PGR5/PGRL1-mediated CEF is considered to be the main pathway in higher plants [13], but how exactly PGR5 and PGRL1 contribute to CEF remains a matter of debate [14].

Chloroplasts also harbor a wide variety of small regulatory proteins, named thioredoxins (Trxs), which allow chloroplast metabolism to be modulated in accordance with light availability. They reduce their target enzymes through thiol–disulfide exchange reactions, using electrons obtained from photo-reduced ferredoxin (Fd) via Fd-dependent thioredoxin reductase (FTR), and they do so in a fast and reversible manner that is light-dependent. Typical TRXs in the chloroplast can be classified into types *f*, *m*, *x*, *y* and *z* [15,16,17]. In addition, chloroplasts possess a second thioredoxin system—NTRC, an NADPH-dependent thioredoxin reductase (NTR) fused to a thioredoxin domain [18,19]. NTRC is reduced by NADPH and, in contrast to the Fd-FTR-Trx system, it also works in the dark. The two redox systems are involved in the regulation of common processes [20,21,22,23,24] and Arabidopsis plants in which both systems are inactivated by mutation show extremely severe retarded growth phenotypes [25,26], indicating that the two systems operate in a concerted manner. Indeed, it has recently been shown that the functions of NTRC and Fd-FTR-Trxs are integrated through redox regulation of the 2-Cys peroxiredoxins (Prxs) [27,28]. NTRC maintains the reductive capacity of Trxs by keeping the chloroplastic 2-Cys Prxs reduced; conversely, in the absence of NTRC, oxidized 2-Cys Prxs receives electrons from Trxs, such that other targets of Trxs become more highly oxidized. Thus, the *ntrc* mutant can be partially rescued by reducing the supply of 2-Cys Prxs (*ntrc-Δ2cp* mutant) [27].

In fact, NTRC is a very efficient reductant of the 2-Cys Prxs and antioxidant functions have been attributed to it [19,29,30]. In particular, plants devoid of NTRC are sensitive to various abiotic stresses, such as high salinity and drought [18], prolonged darkness [19] and heat [31]. However, the *ntrc* mutant is protected against excess light by the very strong induction of NPQ [32]. This comes about because the γ subunit of the ATP synthase [32,33], as well as the Calvin–Benson cycle enzymes [27], are more highly oxidized in the absence of NTRC, which results in lower proton consumption and hence increased acidification of the thylakoid lumen, which triggers NPQ even at low light intensities [32]. A similarly elevated NPQ is observed in plants that are defective in ATP synthesis, such as the *cgl160* mutant [34]. In contrast, plants defective in PGR5 are deficient in proton gradient formation, which impairs the induction of NPQ [9]. Moreover, in the *pgr5* mutant, the production of ATP is decreased and the lower ATP/NADPH ratio reduces the supply of electron acceptors from PSI, which causes the overreduction of the stroma and P700, and consequently, triggers photo-inhibition [4,35]. In consequence, *pgr5* plants are more sensitive to light stress and show a lethal phenotype under fluctuating light conditions, because they cannot adjust their photosynthetic performance to the changes in light intensity [36,37]. 

The various photosynthetic electron pathways are closely interconnected. For instance, it has already been reported that plant mutants with decreased LEF can rescue the lethal phenotype of *pgr5* under fluctuating light [37,38]. To further investigate the interplay between LEF and CEF, we generated the double mutants *pgr5 ntrc* and *pgr5 cgl160*. With this genetic approach, we aimed to study the effects of combining the defect in chloroplast ATPase (cpATPase) activity present in *ntrc* and *cgl160* mutants (both of which exhibit elevated NPQ) with the defect in CEF in the *pgr5* mutant (which suppresses NPQ) on plant growth and photosynthesis. Our intention was to increase the impaired NPQ in the *pgr5* mutant by introducing the *ntrc* and *cgl160* mutations, and thus to recover the growth of *pgr5* under fluctuating light.

## 2. Materials and Methods

### 2.1. Plant Material and Growth Conditions

*Arabidopsis thaliana* wild type (Col-0 and Col-5) and mutant plants were grown on soil at 100 µmol photons m^−2^ s^−1^ under different photoperiods (8 h light/16 h darkness (short-day, SD), 12 h light/12 h darkness or 16 h light/8 h darkness (long-day, LD)), under high light ((HL): 500 µmol photons m^−2^ s^−1^, 16 h light/8 h darkness) or under fluctuating light ((FL): cycles of 5 min at 50 μmol photons m^−2^ s^−1^/1 min at 500 μmol photons m^−2^ s^−1^ during the light period, 12 h light/12 h darkness). Temperature (22 °C/20 °C during the day/night cycle) and relative humidity (60%) were strictly controlled under all these conditions. Plants were weighed before flowering in all cases: after 6 weeks in SD, 5 weeks in 12 h/12 h, 3 weeks in LD, 2 weeks in HL and 4 weeks in FL.

The Arabidopsis mutants *pgr5* (*pgr5-1*, S130G point mutation), *pgrl1ab* (SAIL_443E10/SALK_059233), *ntrc* (SALK_012208), *cgl160* (*cgl160-1*, SALK_057229) and *trx m4* (SALK_023810) were previously described ([9,10,18,34,39], respectively). The double mutants *pgr5 ntrc* and *pgr5 cgl160* were obtained by manually crossing the respective parental mutant lines and subjecting the progeny to PCR analysis of genomic DNA. Oligonucleotide sequences used for PCR genotyping are listed in Table S1. The *pgr5* allele was analyzed by amplifying and sequencing the genomic region spanning the region coding for the S130G point mutation in At2g05620/PGR5 [9].

### 2.2. Chlorophyll a Fluorescence Measurements

In vivo chlorophyll *a* fluorescence was monitored with the Imaging PAM chlorophyll fluorimeter (Imaging PAM, M-Series; Walz, Effeltrich, Germany). Plants were dark-adapted for 30 min and induction–recovery curves were plotted after exposure to actinic light at 80 or 390 μmol photons m^−2^ s^−1^ for 10 min, followed by 8 min of darkness. Saturating pulses (0.8 s duration) were applied every 60 s to determine the different parameters, which were calculated by the Imaging PAM software according to the equations in [40]. 

### 2.3. Protein Extraction and Immunoblot Analysis

Rosette leaves (50 mg fresh weight) were ground in liquid nitrogen and homogenized in 500 µL of 2× Tricine buffer containing 8% (*w/v*) SDS, 24% (*w/v*) glycerol, 15 mM DTT and 100 mM Tris/HCl (pH 6.8). The homogenate was incubated for 5 min at 70 °C and centrifuged for 10 min at 13,000× *g*. Solubilized leaf proteins corresponding to 3 mg fresh weight were loaded onto 10% Tricine–SDS–PA gels [41]. Resolved proteins were transferred to polyvinylidene fluoride (PVDF) membranes (Immobilon-P; Millipore, Burlington, MA, USA) using the BioRad blotting system Trans-Blot Turbo (Hercules, CA, USA). PVDF membranes were blocked with 1% (*w/v*) milk in TBS-T (10 mM Tris, pH 8.0, 150 mM NaCl, and 0.1% Tween 20) and probed with antibodies against NTRC (1/1500 dilution, provided by Prof. Cejudo), PGR5 (1/2500 dilution, [9]), PGRL1 (1/10,000 dilution, [10]) and CGL160 (1/10,000 dilution, [34]). Equal loading was verified by staining PVDF membranes with Coomassie Brilliant blue R-250 dye. Signals were visualized with enhanced chemiluminescence using the Pierce™ ECL Western Blotting substrate reagent (ThermoFisher Scientific, Waltham, MA, USA) or the ECL SuperBright (Agrisera, Vännäs, Sweden) in the case of anti-PGR5, and were quantified using the ImageJ software [42].

### 2.4. Alkylation Assays

To determine the in vivo protein redox state, alkylation assays were performed as previously described by Naranjo et al. (2016) using NEM (*N*-ethylmaleimide, ThermoFisher Scientific). Leaves were ground in liquid nitrogen and 10% (*v*/*v*) trichloroacetic acid (TCA) was immediately added to quench thiol oxidation. Samples were incubated on ice for 20 min and then centrifuged at 16,200× *g* and 4 °C for 10 min. The pellets were washed with acetone, resuspended in alkylation buffer (2% (*w/v*) SDS, 50 mM TRIS–HCl pH 7.8, 2.5% (*w/v*) glycerol, and 4 M urea) with 10 mM NEM, and incubated for protein thiol alkylation for 20 min at room temperature. Alkylated samples were subjected to SDS-PAGE and probed using PGRL1 antibody as described above. 

## 3. Results and Discussion

### 3.1. Growth and Photosynthetic Performance of the pgr5 ntrc Double Mutant under Different Light Conditions 

**Figure 1:**
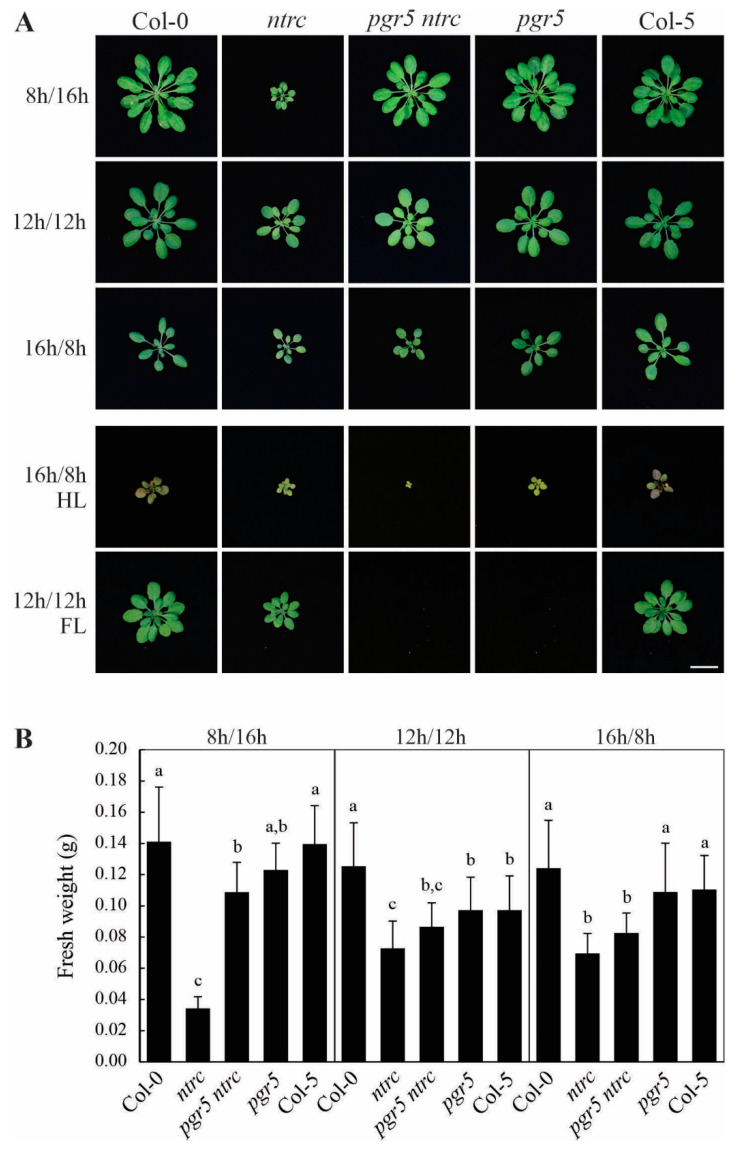
The retarded growth phenotype of *ntrc* is suppressed by *pgr5* under short-day conditions. (**A**) Growth phenotypes of wild type (Col-0 and Col-5) and mutant (*ntrc*, *pgr5* and *pgr5 ntrc*) plants grown on different photoperiods (100 µmol photons m^−2^ s^−1^): 8 h light/16 h dark (short-day, 6-week-old plants), 12 h light/12 h dark (5 weeks old), 16 h light/8 h dark (long-day, 3 weeks old), and under lighting conditions: high light (HL, 500 µmol photons m^−2^ s^−1^, 2 weeks old) and fluctuating light (FL, 50 µmol photons m^−2^ s^−1^ for 1 min and 500 µmol photons m^−2^ s^−1^ for 5 min, 5 weeks old). The scale bar at the bottom indicates 1 cm. (**B**) Fresh weight averages of plants grown as in (**A**). Error bars correspond to SDs for *n* ≥ 10. Letters indicate significant differences, as assessed with the Tukey test and a confidence interval of 95%.

To compensate for the deficit of electron acceptors caused by the *pgr5* mutation, several approaches have been successfully employed. These include adding methyl viologen (MV) [9] or overexpressing *Physcomitrella patens* flavodiiron protein genes in the *pgr5* mutant [38]. In addition, decreasing LEF and lowering electron donation to PSI complement the *pgr5* phenotype, as it has been shown using DCMU [37], by introducing a defect in the oxygen-evolving complex (in the *Δ5 pgr5* mutant) [13], and by downregulating the activity of the cyt *b_6_f* complex (in the *pgr5 pgr1* mutant) [38]. Therefore, we hypothesized that decreasing LEF by increasing NPQ in a *pgr5 ntrc* double mutant would suppress the lethal phenotype of *pgr5* under fluctuating light—owing to the absence of NTRC [32]. However, the *pgr5 ntrc* double mutant, like the *pgr5* single mutant, was incapable of surviving under fluctuating light (Figure 1A), indicating that the *ntrc* mutation does not suppress the effects of *pgr5* in this regard. In marked contrast, the *pgr5* mutation largely suppresses the growth defect associated with the *ntrc* mutation under short-day lighting conditions (Figure 1A,B).

**Figure 2:**
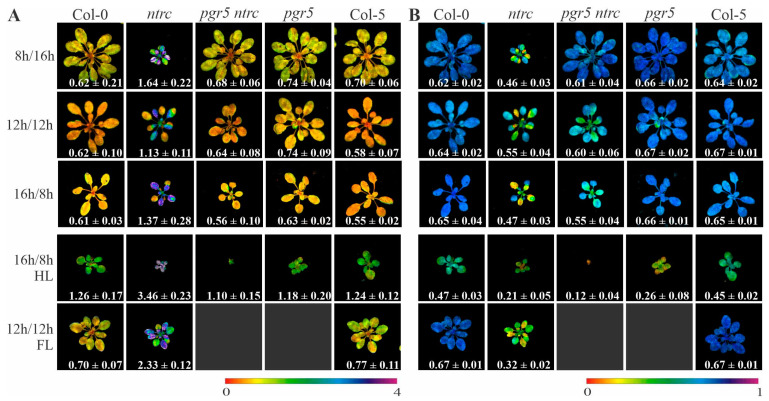
Steady-state non-photochemical quenching (NPQ) and effective quantum yield of photoScheme 5. and/or NTRC, as well as WT (Col-0 and Col-5) control plants, grown under different lighting conditions. (**A**) NPQ of wild type and mutant (*ntrc*, *pgr5* and *pgr5 ntrc*) plants grown on different photoperiods (100 µmol photons m^−2^ s^−1^): 8 h light/16 h dark (short-day, 6 week-old plants), 12 h light/12 h dark (5 weeks old), 16 h light/8 h dark (long-day, 3 weeks old), and under different lighting conditions: high light (HL, 500 µmol photons m^−2^ s^−1^, 2 weeks old) and fluctuating light (FL, 50 µmol photons m^−2^ s^−1^ for 1 min and 500 µmol photons m^−2^ s^−1^ for 5 min, 5 weeks old), after 9 min of illumination with actinic light (80 µmol photons m^−2^ s^−1^). (**B**) Φ_II_ corresponding to the plants in (**A**). NPQ and Φ_II_ averages ± SD values (*n* ≥ 4) are indicated; signal intensities correspond to the colour scale at the bottom of the panel.

Plants devoid of NTRC show a stunted and pale phenotype, and are hypersensitive to diverse abiotic stresses [18], but not to high light stress [32]. In addition, it has been shown that, in the *ntrc* mutant, NPQ is enhanced and the effective quantum yield of photosystem II (Φ_II_) is diminished, which protects *ntrc* against high light but also impairs its growth under low light or short-day conditions ([32] and Figure 1 and Figure 2). Interestingly, the introduction of the *pgr5* mutation into the *ntrc* background decreased steady-state NPQ to WT-like levels under all the conditions analyzed—including high light (Figure 2A and Figure S1A). However, this effect was not always associated with the recovery of Φ_II_ and growth (Figure 1, Figure 2B and Figure S1B), implying that the WT steady-state NPQ level is not sufficient to cope with light stress in the absence of NTRC and PGR5. For instance, under short-day conditions Φ_II_ and growth rate of *pgr5 ntrc* were very similar to those of WT plants, whereas under long-day conditions, the *pgr5 ntrc* mutant failed to reach WT-like Φ_II_ values, and its growth rate resembled that of the *ntrc* single mutant (Figure 1 and Figure 2B). Under high light, the combination of *pgr5* with *ntrc* was virtually lethal (Figure 1A and Figure 2B). These results suggest a role for NTRC in photoprotection beyond negatively influencing NPQ induction—possibly via ROS scavenging through the reduction of 2-Cys Prxs—that is usually masked by the high level of NPQ. 

### 3.2. NTRC-Dependent NPQ Induction in the Absence of PGR5 

In previous studies, the increase in steady-state NPQ in the absence of NTRC was attributed to a lower lumenal proton efflux due to impaired Calvin–Benson cycle and ATP synthase activities, both of which amplify the proton gradient across the thylakoid membrane [27,32,33]. However, here we clearly show that the additional steady-state NPQ induced by the absence of NTRC requires PGR5. 

**Figure 3:**
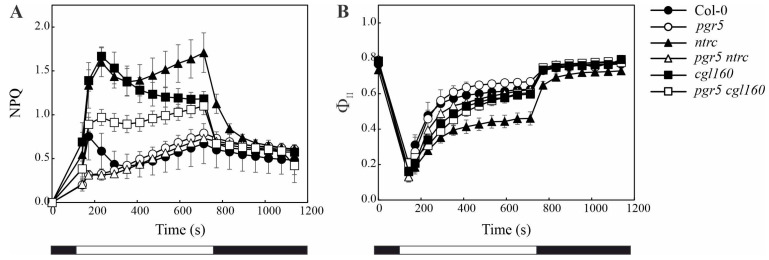
Non-photochemical quenching (NPQ) and photosynthetic performance in *ntrc*, *pgr5* and *cgl160* mutants. (**A**) NPQ induction and recovery was monitored in dark-adapted Col-0, *pgr5*, *ntrc*, *pgr5 ntrc*, *cgl160* and *pgr5 cgl160* plants grown for 6 weeks under short-day conditions. Plants were illuminated for 10 min with 80 μmol photons m^−2^ s^−1^ actinic light (white bar), followed by a dark period of 6 min (black bar). (**B**) Photosystem II quantum yield (Φ_II_) corresponding to the measurements shown in (**A**). Averages of at least six replicates are shown. Error bars represent standard deviations. Col-5 (data not shown) behaves like Col-0.

In addition to steady-state NPQ, we also measured the kinetics of NPQ induction upon a dark-to-light transition, which serves as a proxy for CEF activity [9,10]. The transient induction of NPQ in *ntrc* is more than doubled compared to WT (Figure 3A) and its relaxation results in the elevated steady-state NPQ levels described above (see Figure 2A). In the *pgr5* mutant, NPQ induction is much weaker than in WT and this has been ascribed to the lack of PGR5/PGRL1-dependent CEF. The transient induction and relaxation of NPQ seen in the *pgr5 ntrc* mutant is very similar to that of the *pgr5* single mutant (Figure 3A), suggesting that the elevated transient NPQ found in the *ntrc* mutant itself depends on a functional PGR5 protein. As a control, we used the *cgl160* mutant, which is impaired in chloroplast ATPase activity and therefore displays an impaired proton motive force (pmf) and an elevated NPQ [34]. Although the increase in transient NPQ induction seen in *cgl160* is very similar to that of *ntrc*, the combination of the *cgl160* mutation with *pgr5* reduced the transient NPQ induction to WT but not *pgr5* levels, and almost no relaxation of NPQ was seen at later time points. These results indicate that the enhancement of both steady-state and transient NPQ in *ntrc* depends on PGR5, while this is not the case in the *cgl160* mutant. Consequently, owing to the loss of the functional PGR5 protein, *pgr5 ntrc* lines behave like *pgr5* with respect to the expression of NPQ. Moreover, in the absence of PGR5, the *cgl160* mutant with a defective chloroplast ATPase still displays transient and steady-state NPQ values that are much higher than those in the *pgr5* single mutant, implying that this residual elevated NPQ is specific to the cpATPase defect, and is not related to PGR5/PGRL1-dependent CEF. Indirectly, this also implies that the elevated (steady-state and transient) NPQ observed in plants that lack NTRC is not caused by impaired cpATPase function, but can be explained by the altered activity of PGR5 alone.

In the *pgr5 cgl160* mutant (Figure S2), the enhanced level of transient NPQ relative to WT was associated with Φ_II_ values that were lower than in WT (Figure 3A,B), indicating that the *pgr5* mutation can restore this component of photosynthesis in the *ntrc* background, but not in the *cgl160* background. In line with this, *pgr5 cgl160* plants remain smaller than either of the two parental lines *pgr5* and *cgl160* under all conditions analysed (Figure S3). Interestingly, even though *pgr5 cgl160* plants are capable of inducing NPQ to levels that are higher than in WT, at least under short-day conditions, these plants are unable to grow under fluctuating light (Figure 3A and Figure S3). These data suggest that the high level of NPQ during the high light period is not decisive for survival under fluctuating light, but possibly its rapid recovery during the low light period is. Moreover, a deficit in ATP synthesis resulting from the absence of the cpATPase assembly factor CGL160 possibly aggravates the stromal overreduction of the *pgr5* mutant, increasing the deficit of electron acceptors and the photodamage of P700.

### 3.3. PGRL1 Dimer Formation and Protein Content in the Absence of NTRC

**Figure 4:**
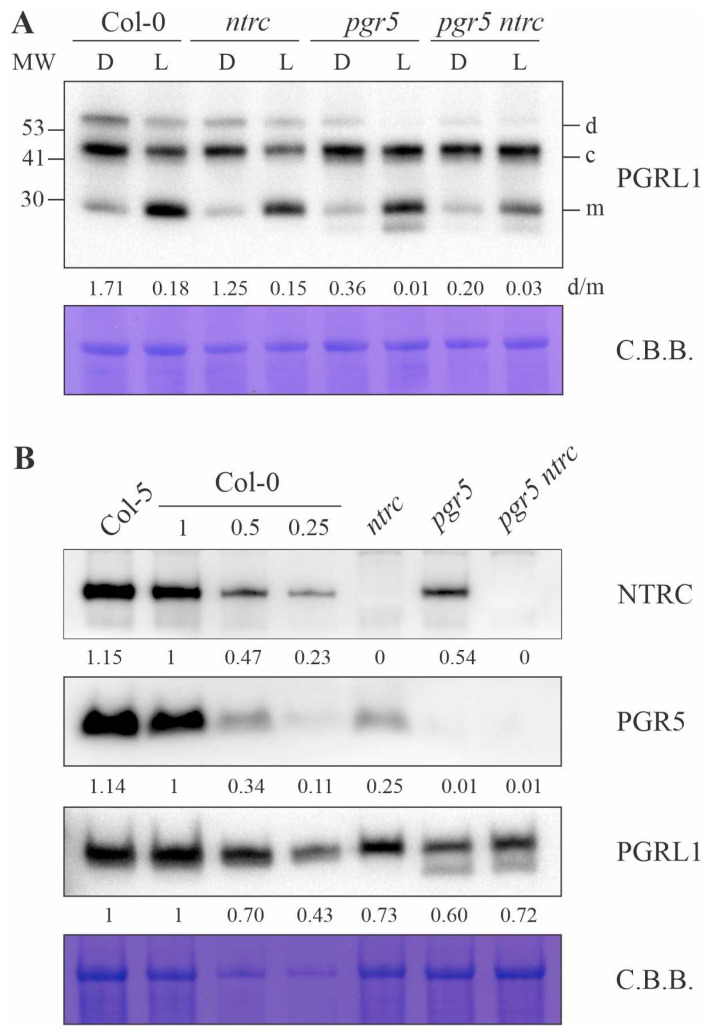
PGRL1 dimer formation and protein accumulation in the absence of NTRC and PGR5. (**A**) Total leaf proteins were obtained from Col-0, *ntrc*, *pgr5* and *pgr5 ntrc* after 6 weeks of growth under short-day conditions, precipitated with TCA and alkylated using NEM at the end of the dark period (dark, D) and after a 30-s exposure to light (L, 100 μmol photons m^−2^ s^−1^). Samples were fractionated by SDS-PAGE and subjected to immunoblotting using PGRL1-specific antibody. MW stands for molecular weight scale (KDa). d, c and m indicate dimer, complex and monomer forms of PGRL1, respectively. A representative blot from three experiments is presented, and the ratio of PGRL1 dimers to monomers (d/m), based on the quantification of band intensities, is shown below the panel. (**B**) Aliquots of total leaf proteins from Arabidopsis wild type (Col-5 and Col-0), *ntrc* and *pgr5* single mutants and *pgr5 ntrc* double mutant after 6 weeks of growth under short-day conditions were fractionated by SDS-PAGE and subjected to immunoblotting using NTRC-, PGR5- or PGRL1-specific antibodies. Decreasing amounts of Col-0 were loaded. Representative blots from three experiments are presented, as well as the values corresponding to the quantification of the intensity of each band relative to Col-0 100%. In both (**A**,**B**), protein samples were adjusted according to fresh weight, and PVDF membranes were stained with Coomassie brilliant blue (C.B.B.) to show protein loading.

Our results indicate that elevated NPQ in *ntrc* depends on PGR5, which implies that in WT plants the NTRC protein has a negative effect on PGR5 activity. This raises the question of how this effect occurs. A physical interaction between NTRC and PGR5, but not PGRL1, has already been reported [43], although neither of the latter proteins appears as an NTRC interactor in pull-down experiments performed in a more recent study [24]. However, PGRL1 is much more likely to be the target of the redox regulation of PGR5/PGRL1-dependent CEF, since it contains six conserved cysteines (Cys), while PGR5 has only one. Indeed, it has recently been shown that the single Cys in PGR5 is dispensable for CEF in Chlamydomonas [44], while in vitro [11] and in vivo experiments [45,46] have confirmed the redox regulation of PGRL1 by Trxs. Taken together, these considerations are compatible with the direct redox regulation of PGRL1 by NTRC to decrease CEF activity—and consequently NPQ. Therefore, we performed in vivo alkylation assays using NEM to analyze the redox dependence of complex formation by PGRL1 in WT and mutant plants after a dark-to-light transition. In fact, PGRL1 was reduced after the dark-to-light transition, with the active monomeric form (m) being more abundant in the light, while the dimer (d, inactive form) was more abundant in the dark and required PGR5 (Figure 4A), corroborating previous results [11,45]. However, we did not observe any significant difference between the dimer/monomer ratios of PGRL1 in *ntrc* and WT plants, either in the dark or after a 30-s exposure to light (Figure 4A), such that an altered PGRL1 dimer/monomer ratio can most likely be excluded as the cause for altered CEF activity in the mutant. Nevertheless, it is necessary to test more light conditions with different exposure times and intensities to definitively rule out this possibility.

It has also been shown that Trx m4 forms a complex with PGRL1 and inactivates it [46]. Indeed, phenotypic effects of *trx m4* [46,47], but not of other thioredoxin mutants like *trx f1f2* [48], can be suppressed by *pgr5*. Multiple sources of evidence support the notion that chloroplast Fd-FTR-Trxs and NTRC act in a concerted fashion, with 2-Cys Prxs serving as connecting links between the two redox systems [27]. Therefore, another possibility is that NTRC influences PGR5/PGRL1-dependent CEF through Trx m4, with NTRC being required for the formation of the PGRL1-Trx m4 complex. However, we could not unambiguously clarify this, since, in our hands, a band (denoted by “c”) with the size of the described PGRL1-Trx m4 complex (40 kDa) was not only present in *ntrc* (Figure 4A) plants, but also in the *trx m4* mutant used as a negative control (Figure S4). Moreover, the intensity of the “c” band changed only a little, if at all, among genotypes and conditions, arguing against a regulatory role of the represented complex in PGRL1 activity.

Interestingly, some photosynthetic-related proteins such as PsaA and PsbO accumulated less in *pgr5* (about 75% and 60% of WT level, respectively) and *ntrc* (both around 40% of WT) mutants, and both were recovered in the *pgr5 ntrc* double mutant (90% and 66% of WT, respectively) (Figure S5). Moreover, the decrease in the NTRC and PGR5 proteins was especially pronounced in the *pgr5* and *ntrc* backgrounds, respectively (Figure 4B and Figure S5B). While the steady-state level of PGRL1 fell to 73% of WT in the *ntrc* mutant, amounts of PGR5 dropped to 25% (Figure 4B) [43]. At first sight, this seems to be at variance with the increased activity of PGR5 that we propose in the absence of NTRC. However, if PGR5 activity is no longer inhibited in the absence of NTRC, then lowering its abundance at the protein level might be necessary to avoid further harmful effects. Accordingly, the enhanced NPQ seen in the *ntrc* mutant might be the result of the enhanced activity of the remaining 25% of PGR5 proteins relative to WT (and PGRL1 to 70% of WT). Intriguingly, NTRC protein levels were also decreased in the *pgr5* mutant to about 50% (Figure 4B). Since the accumulation of PGR5 and PGRL1 is interdependent [10,11], it is not possible to unambiguously assign each drop in abundance of a given protein specifically to another one in this three-protein set, but there is clearly a two-way type of interdependence of protein levels between PGR5/PGRL1 and NTRC.

## 4. Conclusions

We have previously reported that NTRC influences NPQ, probably through the activation of chloroplast metabolism and enhanced thylakoid proton consumption [27,32]. However, by generating and analysing the *pgr5 ntrc* double mutant, we have demonstrated here that the high NPQ observed in the absence of NTRC is mediated by PGR5 and must be a consequence of the deregulation of its activity. In line with this, PGR5 levels are reduced in the *ntrc* mutant, probably to compensate for this deregulation. Therefore, we propose that in WT plants, NTRC controls PGR5/PGRL1-dependent CEF activity. Further studies will be needed to clarify the mechanism, which could involve the direct regulation of PGR5, PGRL1 or a third player, either directly by NTRC, or indirectly through other thioredoxins (such as Trx m4) and 2-Cys Prxs. In addition, the *pgr5 ntrc* mutant uncovered the role of NTRC beyond controlling NPQ induction in the protection of plants against high light stress, possibly through ROS scavenging via 2-Cys Prxs.

## Data Availability

Data is contained within the article or supplementary material.

## Supplementary Materials

The following are available online at https://www.mdpi.com/article/10.3390/antiox10060900/s1, Figure S1: non-photochemical quenching (NPQ) and effective quantum yield of photosystem II (Φ_II_) in long-day plants lacking PGR5 and NTRC, Figure S2: *pgr5 cgl160* double mutant, Figure S3: *pgr5* impairs the growth of *cgl160* under different light conditions, Figure S4: PGRL1 complexes formation, Figure S5: accumulation of representative photosynthesis-related proteins in *ntrc*, *pgr5* and *pgr5 ntrc* plants compared to wild type (Col-0 and -5), Table S1: oligonucleotide sequences used for PCR genotyping.
