# Supplementary material for: NTRC Effects on Non-Photochemical Quenching Depends on PGR5
Source: Antioxidants (Basel). 2021 Jun 3;10(6):900. doi: 10.3390/antiox10060900 (PMC8229092; doi:10.3390/antiox10060900)
Supplement: Supplementary file 1 [file antioxidants-10-00900-s001.zip › antioxidants-1202801-supplementary.pdf]

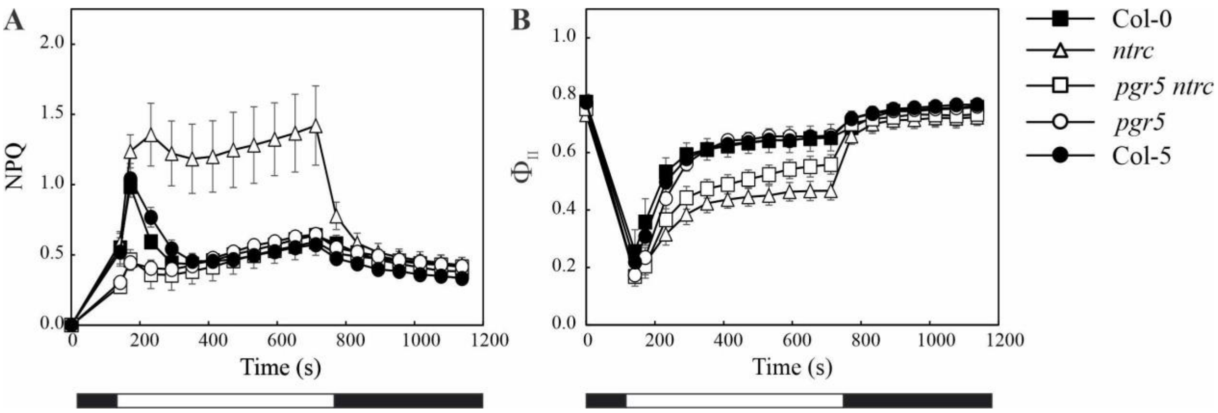

Figure S1. Non-photochemical quenching (NPQ) and effective quantum yield of photosystem II (Φ_{II}) in long-day plants lacking PGR5 and NTRC. (**A**) NPQ monitored in dark-adapted Col-0, Col-5, *ntrc*, *pgr5* and *pgr5 ntrc* plants grown for 3 weeks under long-day conditions. Plants were illuminated for 10 min with 80 µmol photons m^{-2} s^{-1} actinic light (white bar), followed by a dark period of 6 min (black bar). (**B**) Φ_{II} corresponding to the measurements shown in (**A**). Averages of at least four replicates are shown. Error bars represent standard deviations.

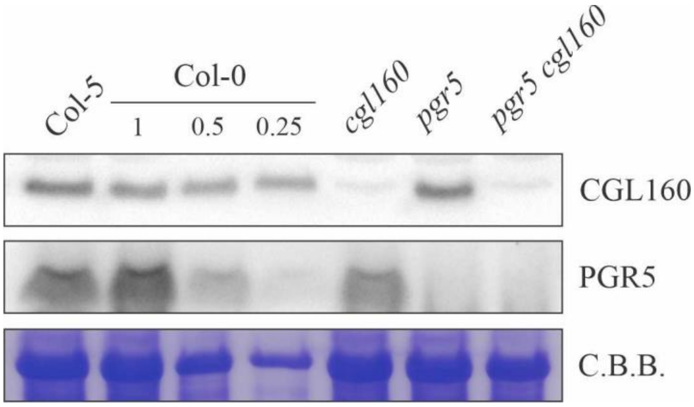

Figure S2. The *pgr5 cgl160* double mutant. Aliquots of total leaf proteins from Arabidopsis wild-type (Col-5 and Col-0), *cgl160* and *pgr5* single mutants and the *pgr5 cgl160* double mutant after 6 weeks of growth under short-day conditions were fractionated by SDS-PAGE and subjected to immunoblotting using CGL160- or PGR5-specific antibodies. Decreasing amounts of Col-0 were loaded. Protein samples were adjusted according to fresh weight and PVDF membranes were stained with Coomassie brilliant blue (C.B.B.) to show protein loading.

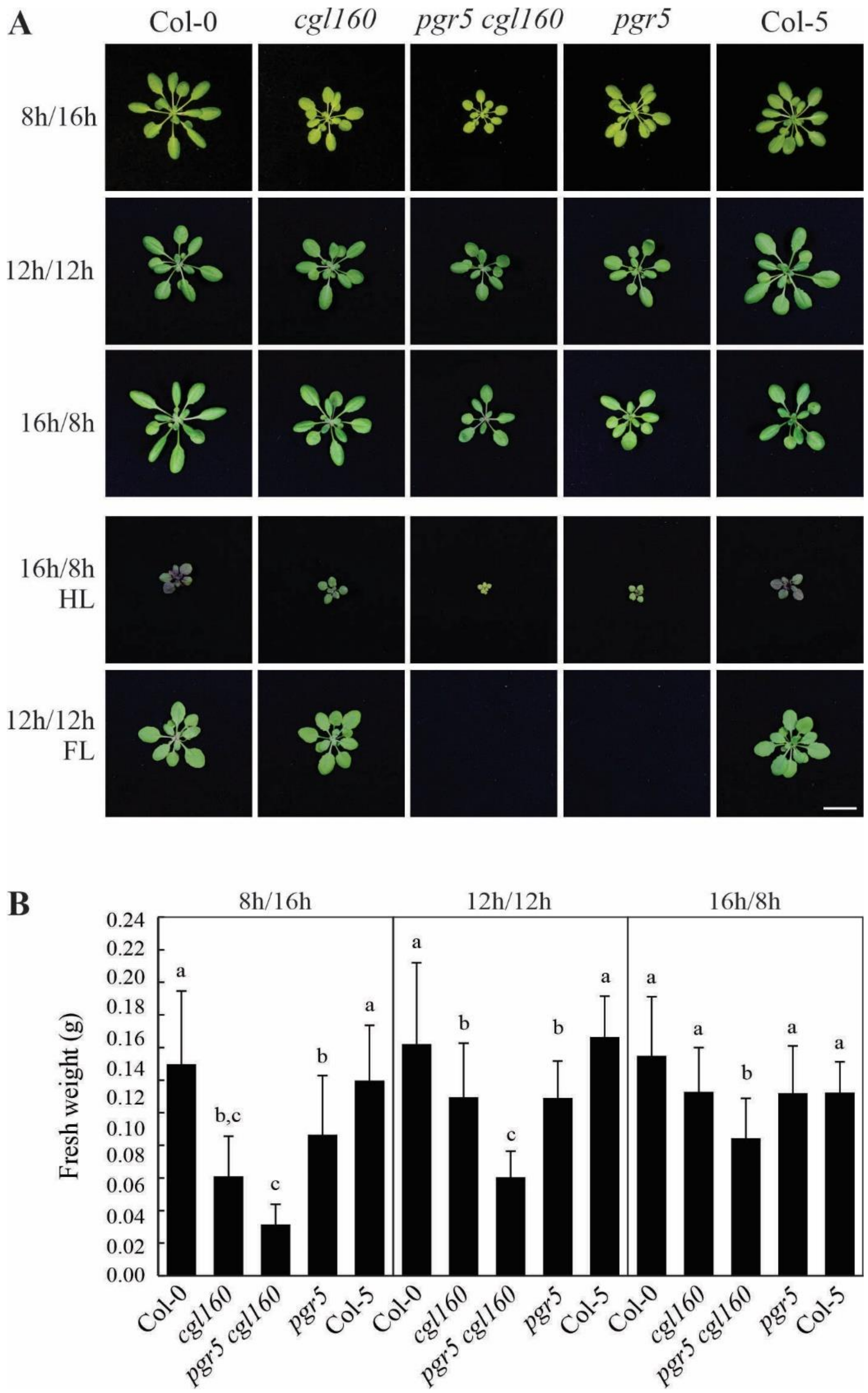

Figure S3. The *pgr5* mutation impairs the growth of *cgl160* plants under different lighting conditions. (**A**) Growth phenotype of wild-type (Col-0 and Col-5) and mutant (*cgl160*, *pgr5* and *pgr5 cgl160*) plants on different photoperiods (100 µmol photons m^{-2} s^{-1}): 8 h light / 16 h dark (short-day, 6 week-old plants), 12 h light / 12 h dark (5 weeks old), 16 h light / 8 h dark (long-day, 3 weeks old) and lighting conditions: high light (HL, 500 µmol photons m^{-2} s^{-1}, 2 weeks old) and fluctuating light (FL, 50 µmol photons m^{-2} s^{-1} for 1 min and 500 µmol photons m^{-2} s^{-1} for 5 min, 5 weeks old). The scale bar at the bottom indicates 1 cm. (**B**) Fresh weight averages of plants grown as in (**A**). Error bars correspond to SDs for $n \geq 10$. Letters indicate significant differences as assessed with the Tukey test and a confidence interval of 95%.

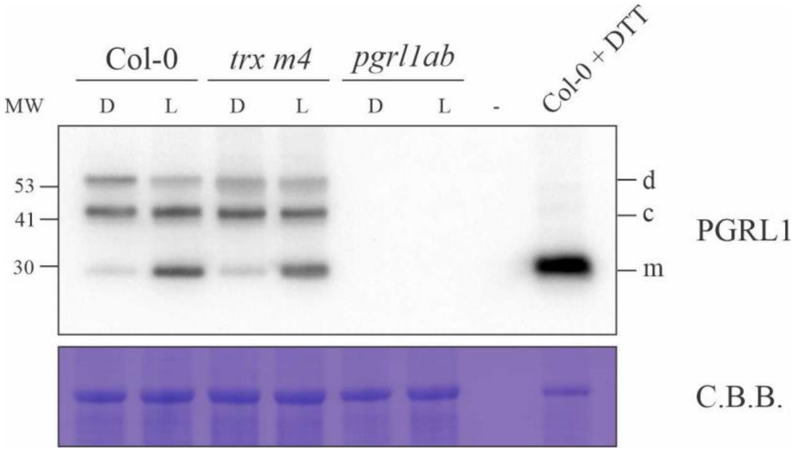

Figure S4. Formation of PGRL1 complexes. Total leaf proteins obtained from Col-0, *trx m4* and *pgrl1ab* plants after 6 weeks of growth under short-day conditions were precipitated with TCA and alkylated using NEM at the end of the dark period (dark, D) and after a 30-sec exposure to light (L, 100 μmol photons m^{-2} s^{-1}). Samples were fractionated by SDS-PAGE and subjected to immunoblotting using a PGRL1-specific antibody. Protein samples were adjusted according to fresh weight. PVDF membranes were stained with Coomassie brilliant blue (C.B.B.) to show protein loading. Representative blots from three experiments are presented. MW indicates the molecular weight scale (KDa). d, c and m indicate dimer, complex and monomer forms of PGRL1, respectively.

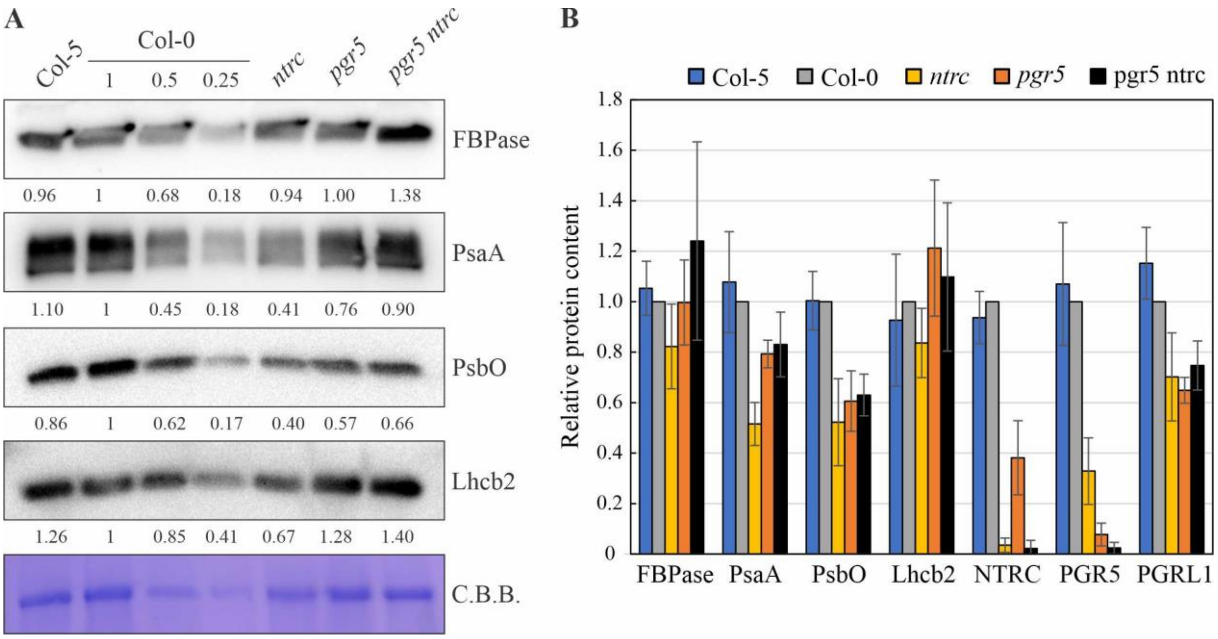

Figure S5. Accumulation of representative photosynthesis-related proteins in *ntrc, pgr5* and *pgr5 ntrc* plants compared to wild type (Col-0 and -5). **(A)** Aliquots of total leaf proteins from Arabidopsis wild-type (Col-5 and Col-0), *ntrc* and *pgr5* single mutants, as well as the *pgr5 ntrc* double mutant after 6 weeks of growth under short-day conditions were fractionated by SDS-PAGE (10% Tris-Glycine gels) and subjected to immunoblotting using FBPase-, PsaA-, PsbO- or Lhcb2-specific antibodies (1/5000 dilution, Agrisera). Decreasing amounts of Col-0 were loaded. Representative blots from three experiments are presented, as well as the values corresponding to the quantification of the intensity of each band relative to Col-0 (Col-0 = 100%). Protein samples were adjusted according to fresh weight, and PVDF membranes were stained with Coomassie brilliant blue (C.B.B.) to show protein loading. **(B)** Average of the protein quantification of three different membranes as shown in (A) and Figure 4B, relative to Col-0 (Col-0 =100%) (relative protein content). Error bars correspond to SDs.

Table S1. Oligonucleotide sequences used for PCR genotyping.

Locus	Gene	Primer (5' to 3')	Notes
At2g05620	*PGR5*	LP: GGTGTAAGTCCAAGCAAGA<br>RP: CGGATTAAGAGCTGATGTTG	
At2g41680	*NTRC*	LP: TCACCAACATGTGGCCC<br>RP: TTCTTCATCTTCACACCCGA	
At2g31040	*CGL160*	LP: AAGTTAAGATTCCATTTTCGCATC<br>RP: TCCCTAAACATCACATCCTGC	
At4g22890	*PGRL1A*	LP: CCAAAGAAGGAGGTGTTTTCC<br>RP: CAAGAGTTTCTCCAAGCGTTG	
At4g11960	*PGRL1B*	LP: GTTTGGGAACACAGTGGCTT<br>RP: ATCAAGGAGGTCCACAAGTCT	
At3g15360	*Trx m4*	LP: AGGATTCATAGGGAGTGG<br>RP: TTTTCTCCAACGTCTCTCT	
-	-	LB3: TAGCATCTGAATTTCATAACCAATCTCGATACAC	T-DNA left border primer for SAIL lines
-	-	LBb1.3: ATTTTGCCGATTTCGGAAC	T-DNA left border primer for SALK lines
